# Analysis of nutritional adequacy of local foods for meeting dietary requirements of children aged 6-23 months in rural central Tanzania

**DOI:** 10.1186/s13690-017-0226-4

**Published:** 2017-08-17

**Authors:** Jofrey Raymond, Morris Agaba, Clara Mollay, Jerman W. Rose, Neema Kassim

**Affiliations:** 1 School of Life Science and Bioengineering, Nelson Mandela African Institution of Science and Technology (NM-AIST), P.O. Box 447, Arusha, Tanzania; 2 SolBridge International School of Business, Woosong University, Daejeon, Republic of Korea

**Keywords:** Child nutrition, Nutritional security, Feeding practices, Local foods, Tanzania

## Abstract

**Background:**

Under nutrition remains a serious problem among children in Sub-Saharan Africa. Analysing how diets composed of local foods could achieve nutritional goals for infants and young children in low-income settings is essential. The objective of this study was to analyse how local foods can be used rationally and to what extent these foods can be supplemented to achieve nutrient requirements for children aged 6 – 23 months in resource-poor settings.

**Methods:**

A cross-sectional study was carried out to estimate dietary intakes of 400 children aged 6-23 months using a 12-h weighed dietary record, 24-h dietary recalls, and 7-days food records. Anthropometric measurements on each subject were also taken. Analyses were done to establish the level of nutrient intake, and nutritional status of the study population using Microsoft Excel 2013 and ProPAN software version 2.0.

**Results:**

The results showed that the prevalence of stunting, wasting and underweight for children aged 6–23 months was 30–41%, 1.5–3% and 4–9%, respectively. In addition, the results showed that diets that were consumed by the subjects comprised of local foods met vitamin A, vitamin C, protein and energy requirements for children aged 6–23 months. However, the extent of deficit in iron, zinc and calcium in baseline diets was large and difficult to meet under the existing feeding practices.

**Conclusions:**

The study shows that local foods in the study area have a potential to achieve recommended dietary intakes of some essential nutrients, and that interventions are needed to meet the required amount of iron, zinc and calcium for children aged 6–23 months. The interventions we propose here may encourage changes in traditional feeding habits and practices of the target population. Possible intervention options are (1) supplementation of local foods with nutrient-dense foods that are not normally consumed in the locality (2) providing new avenues for increasing the production and wide consumption of local nutrient-dense foods, or optimizing the way local diets are constituted so as to achieve nutrient recommendations for infants and young children.

## Background

Malnutrition prevents infants and young children from growing to their full genetic potential and it remains a major problem in Sub-Saharan Africa (SSA) despite the presence of an array of local foods that could ensure nutritional security [1]. Among children in SSA; more than 38.5% (>56 million) suffer chronic undernutrition, nearly 80% have iron deficiency anemia, 50% are at risk of blindness due to vitamin A deficiency, and more than 4 million babies are born underweight annually [2].

Under nutrition can impair physical and cognitive development of children, especially when it occurs during the first 1000 days of life as measured from conception. This period is also considered as a critical window for preventing the effects of under nutrition [3, 4]. Under nutrition at any stage also weakens the ability to fight potentially deadly infectious illnesses [5–7]. In a long term, early-childhood undernutrition leads to shorter adult stature, reduced school achievement and lower economic productivity in adulthood [3, 8]. Furthermore, under nutrition at young age contributes more to a country’s overall disease burden than under nutrition in old children [9].

Most of developing countries, international agencies, non-governmental organizations (NGOs) are currently promoting food based approaches incorporating locally available foods as sustainable solution for combating nutritional insecurity [10, 11]. The assumption behind food-based approach is that local foods can be easily accepted by the community and so the approach aligns with normal feeding patterns and cultural food preferences. Another assumption of the food-based approach is that nutrient-dense foods are locally available and accessible by the target population. However, local foods alone may not achieve the recommended nutrient requirements as has been found to be that case by studies in Indonesia, Cambodia, Guatemala and Myanmar where local foods alone could not satisfy nutritional requirements in low income communities [12–15]. Moreover, there can be significant variability of cultures and feeding practices, and the nutrient density profiles of local foods [14] and consequently dietary recommendations should follow local assessment.

Therefore, the goal of this study was to establish the extent to which local foods could be used rationally to provide basic nutritional needs, and the level of supplementation that would be needed to achieve the recommended nutrient intakes of infants in resource-poor rural setting in Tanzania.

Tanzania is a typical sub-Saharan country with a largely rural based population, which is rapidly urbanising. Like other sub-Saharan countries, Tanzania exhibits high cultural diversity and feeding practices. Cereal (maize) is a dominant staple food superimposed on other locally available foods like fish, livestock products, nuts, seeds and vegetables. Sea foods, for instance, are mainly found in the coastal region, plantains and bananas in the northern highlands and western zone, and nuts in the central region of Tanzania.

## Methods

### Site description

The study was conducted in six randomly selected villages out of 59 in Bahi district, central zone of Tanzania. This district was purposively selected because it is located in a semi-arid area with a low productivity ecosystem. Furthermore, the district is in a rural area that is facing combined effects of demographic challenges (rapid growth and change in population characteristics) and climate and weather variability which may affect traditions and the local food production and consumption patterns and consequently impact on nutritional security.

### Study design

A cross-sectional survey was conducted from September to December 2015 during the dry season to assess food consumption patterns and anthropometric measurements of children aged 6–23-months. A research protocol of the present study was approved by the Institutional Research Committee at the School of Life Sciences and Bioengineering, The Nelson Mandela African Institution of Science and Technology (NM-AIST) and a written informed consent was obtained from each participant parent or responsible guardian. All the data was entered into excel spreadsheets and analyses were done using Microsoft Excel 2013 and ProPAN software.

### Subjects and sampling

A sample size of 67 children were randomly selected from each of the six villages selected for the study. The sample size was previously drawn from a list using ProPAN sampling guidelines specific for children under 24 months old at 95% confidence interval (CI) [16]. The inclusion criteria were as follows: (1) that the child was 6 – 23 months of age inclusive (2) the child’s primary caregiver was available and agreed to participate in the study, (3) the child was apparently healthy and not suffering obvious symptoms that may affect his or her dietary intakes and (4) if more than one child in a household met the inclusion criteria 1 – 3, then one was randomly selected.

### Assessment of nutritional status

Duplicate measurements of each child’s weight and recumbent length were recorded using standard anthropometric procedures described by Gibson [17]. Recumbent length was measured using a length board (SECA, precision ±0.1 cm, Model 4,171,721,009, US). The body weight of children was measured using the infant-hanging weighing scale (Globe Universal; precision ±1 g), with a child minimally clothed. The scale was set to zero before each measurement. All measurements were standardized into Z-scores and the World Health Organization (WHO) growth standards were used to calculate anthropometric indices (height-for-age Z-score, HAZ; weight-for-age, WAZ; and weight-for-height WHZ) for children aged 6–23 months. The three indices were used to classify the nutritional states of children as follows: stunted when all three indices had a Z-score less than −2, underweight or wasted when the height-for-age (HAZ), weight-for-age (WAZ) and weight-for-height (WHZ) Z-scores were less than −2 SD and as severely stunted, underweight or wasted if the respective Z-scores were less than −3 SD. [18]. A child was considered within normal range if their respective indices were between -2SD and +2SD.

### Food consumption patterns

Dietary data were collected using 24-h dietary recalls, weighed dietary record (WDR) and 7 days food records described in previous studies [13, 16, 19]. The WDR method was used to collect data on food consumption for 7 days, whereas, 24-h dietary recalls and 7-day food records were used to describe food consumption patterns in 7 days. Food portion sizes were not collected for the 24-h recall and 7-day food records.

A single 24-h recall was done to obtain information on foods and beverages consumed within 24 h before the WDR day. During the interview, mothers were asked to recall all foods and beverages consumed by their infants in the past 24 h. To help identify foods consumed, pictures and food models were shown to the mother or primary caregiver so that she or he could point out the food items. Mothers were also asked to mention other foods and beverages that were not included in our food models and pictures. Primary caregivers were further asked to describe how often foods identified from 24 h recalls were consumed in the past 7 days.

In the WDR method, all foods and beverages consumed by the child were weighed using electronic kitchen scale (CAMRY, Model EK3131, precision ±2 g) and recorded. A researcher was present at household for 12 h of the day to observe and weigh the amount of all foods and beverages served and consumed by the child as well as leftovers. A 12-h recall for all foods and beverages consumed after the observer has left the household was also collected. Care givers were asked to estimate the amount of recalled foods in local cups or utensils, and the estimated amount were then weighed using the real food models. All composite foods and dishes were broken down into their individual ingredients such as added oil, baobab powder, sugar, salt, tomato, carrots, onions etc. The weighed dietary records were collected on all days of the week to account for the effect of any day of the week on dietary intakes of the subjects.

In the case of 7-days food records, a self-administered 7-day food tally was done to obtain information on the frequency of foods and beverages consumed after the WDR day. In this approach, primary caregivers were asked to record all foods and beverages consumed by their children during the 7-day period. For illiterate primary caregivers and those who forgot to record foods consumed, a 12-h recall was performed. Finally, dietary records obtained from primary caregivers were classified based on the rapid changes in dietary patterns across age groups (6–8 months, 9–11 months and 12–23 months inclusive) [20].

### Dietary nutrient intakes

Dietary intakes of nutrients were estimated based on Tanzania food composition tables [21] and United States Department of Agriculture food composition database [22]. In some cases, the amount of foods served and consumed were converted to their raw form using cooked to raw conversion factors so as to match nutrient values in food composition databases [16]. Furthermore, in order to avoid overestimation, nutrient values for the foods consumed in cooked state were adjusted for cooking losses using USDA retention factors [23]. The observed nutrient intakes were compared with the recommended nutrient intakes established by WHO for young children in developing countries [24–26]. Adequate nutrient intake of the children in the study was assumed if 100% of the children meet the proxy EAR. Furthermore, energy intake was estimated and expressed per day, and was also compared to international energy recommendations. Energy recommendations were based on the percentage of children who met their median energy recommendation given breast milk intakes [27]. The amount of breast milk was estimated from values published by WHO in 1998 [28]. Since the recommendation is based on a median, by definition if 50% or more of the children met the recommendation, then we considered that the complete sample met the recommended energy intake.

### Dietary nutrient densities

The energy density (in kilocalories per gram) of the diet consumed in a 12-h period of the day was estimated by adding up the energy consumed in kilocalories from weighed dietary record and divided by the grams of food and liquids consumed in the same time frame as described in the ProPAN tool [16]. Similarly, nutrient densities of diets consumed was calculated by taking a total amount of a particular nutrient consumed dividing by total energy (in kilocalories) consumed during the 12-h of the day multiplied by 100. The results were then compared to the required nutrient densities of complementary foods described in the WHO document for average breastfed young children in developing countries [28]. In estimating energy and nutrient densities of foods, all composite dishes were broken down into their individual ingredients. Each ingredient was then weighed and recorded separately.

### Adequacy of meal frequency

Meal frequency was calculated by dividing the number of breastfed children aged 6–23 months who received solid, semi-solid or soft foods the minimum number of times or more during the previous 24 h with the total number of breastfed children 6–23 months. In this method, the minimum is defined as 2 times meals for breastfed infants 6–8 months and 3 times meals for breastfed infants 9–23 months. And meals include both formal (such as breakfast, lunch and dinner) and informal (such as snacks) meals. Estimation of meal frequency for breastfed children included only non-liquid feeds as described in the WHO guiding principles for assessing infant and young child feeding practices [29].

## Results

### Characteristics and anthropometric indices of subjects

Four hundred infants and young children from 400 predominantly rural households were surveyed in Bahi district in central Tanzania, 50% percent of the children were aged 12–23 months, 25% were aged 9–11 months and 25% were aged 6–8 months. All the children were still being breastfed at the time of the survey. Four children who indicated signs of acute malnutrition were referred to Bahi district hospital and were excluded from subsequent analysis. The anthropometric profiles of the study sample is presented in Table 1. In general and based on the WHO child growth classification [30], the level of stunting among children 6 – 8 months can be characterized as “high prevalence” and stunting among children aged 9 - 23 as “very high prevalence”. The observed prevalence of wasting or underweight children was within acceptable limits or of low prevalence respectively.

**Table 1:** Nutritional status of children between 6 and 23 months in Bahi district expressed as percentages and the corresponding standard deviation

	6-8 months (*n* = 100)		9-11 months (*n* = 100)		12-23 months (*n* = 200)	
Status	Percentage	SD	Percentage	SD	Percentage	SD
Stunting (HAZ scores < −2)	30	0.7	41	0.7	40.5	0.7
Underweight (at Z scores <−2)	9	0.8	8	0.5	4	0.5
Wasting (at Z scores <−2)	3	0.8	3	0.6	1.5	0.6
Overweight (at Z scores <−2)	19	0.8	41	0.5	19	0.5

### Food patterns and nutrient intakes

**Fig. 1:**
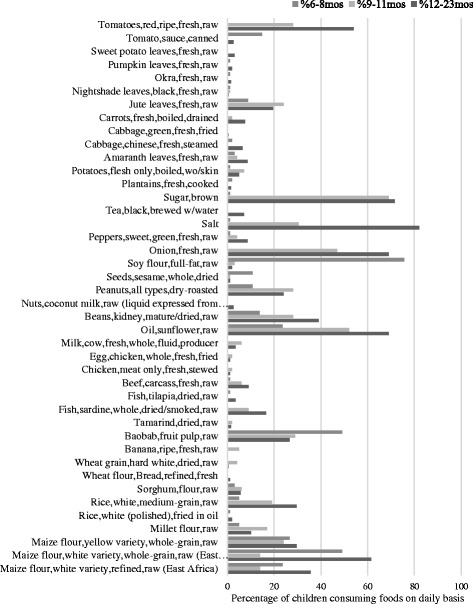
Foods consumed by children aged 6-23 months in percentage

From the dietary survey we identified a total of 33 food items that was used to make food for children in Bahi district. The top 10 most frequently consumed local foods were maize products, sugar, salt, onions, tomatoes, sunflower oil, groundnuts (peanuts), beans, rice and baobab powder. Pulses and seeds (for example, soybeans, sesame seeds and pumpkin seeds), and green leafy vegetables (such as black nightshade, sweet potato leaves and amaranth leaves) were reported less frequently, and foods of animal origin, with exception of dried small fish, were almost absent from the diets. Generally, there was no difference between the age groups in the components of the food basket, however, children aged 12–23 months consumed the higher diversity of identified foods than other age groups (Fig. 1).

The distribution of nutrient intakes and their adequacy for each age group are shown in Table 2. Based on the complementary foods consumed, the estimated average intake of energy achieved the required adequacy for all age groups. Furthermore, the estimated intakes of protein, vitamin A and vitamin C met the adequacy recommended by WHO for all the age groups studied. However, the intake of iron, zinc and calcium for children aged 6 - 8 months, iron and calcium for 9–11 months and iron for 12–23 months did not achieve the recommended adequacy levels.

**Table 2:** Average nutrient intakes per population of children aged 6-23 months

	6-8 months (*n* = 100)		9-11 months (*n* = 100)		12-23 months (*n* = 200)	
Nutrients	Median	Percent of RDI	Median	Percent of RDI	Median	Percent of RDI
Iron (mg)	4.8	23	6.1	29	7.0	58
Zinc (mg)	3.0	60	11.3	226	10.8	166
Calcium (mg)	319.8	61	339.5	65	395.7	113
Vitamin A (μg of RE)	793.2	227	792.9	227	1289.0	322
Vitamin C (mg)	69.5	278	53.1	212	72.5	242
Protein (g)	19.4	213	24.8	258	35.9	331
Energy (kcal)	841.2	306	925.9	206	1110.0	144

### Nutrient densities and adequacy

Table 3 provides information on nutrient densities of children’s complementary diets and their adequacies. The estimated average energy, protein and vitamin C densities of the complementary diets consumed by average breastfed children aged 6–8, 9–11 and 12–23 months met the average desired densities. On the other hand, the average densities of iron, zinc, calcium and vitamin A in diets consumed by infants and young children in all age groups were observed to be lower than the recommended adequacy.

**Table 3:** Average nutrient densities in diets consumed by children aged 6-23 months

	6-8 months (*n* = 100)		9-11 months (*n* = 100)		12-23 months (*n* = 200)	
Nutrients	Median	Percentage	Median	Percentage	Median	Percentage
Iron (mg)	0.66	9	0.66	14	0.75	47
Zinc (mg)	0.48	30	0.53	53	0.56	70
Calcium (mg)	13.03	10	9.70	12	9.48	36
Vitamin A (μg of RE)	1.86	37	2.48	27	4.24	25
Vitamin C (mg)	2.93	293	1.19	119	1.17	106
Protein (g)	1.63	233	2.01	287	2.36	337
Energy (kcal)	3.62	577	3.61	672	3.33	466

### Meal time frequency

The mean frequency results are summarized in Table 4. The results showed that more than 80% of children aged 6–23 months met the recommended meal times a day.

**Table 4:** Average meal time frequency and adequacy for children aged 6-23 months

Age group	Mean meal frequency	SD	Percentage adequacy (%)
6-8 months (*n* = 100)	2.8	0.2	140
9-11 months (*n* = 100)	3.2	0.3	107
12-23 months (*n* = 200)	3.3	0.4	110

## Discussion

The results of the present study suggest that feeding practices is a key factor that significantly affects effectiveness of local foods in providing adequate nutrition for vulnerable infants and young children. Based on the estimated nutrient densities of local diets and individual nutrient intakes, the existing dietary practices may not achieve the recommended intakes of iron, zinc and calcium for children aged 6–23 months despite the presence of nutrient-dense foods. This is because majority of consumers in poor households do not take into account the full costs and benefits of a balanced diet. Most of the households rely much on carbohydrate rich foods (cereal products) as the main component in their diets (Fig. 1 **)**. The reason is probably due to local traditional notions of what constitutes food, for instance, food is maize [31]. Majority of consumers in typical African households regard a meal as food if it is a cereal based meal. Furthermore, typical African households are interested on abetting hunger as a singularity, and so nutrition is rarely considered or factored into diets. As a result they miss opportunities around other nutrient-rich food stuffs for example, pulses, seeds and animal source foods. All these factors affect the nutritional status of an individual child and may eventually lead to chronic under-nutrition especially, among low income urban and most rural households.

Energy and nutrient intakes can provide evidence to the quality of dietary practices in the community. Based on the WHO/FAO/UNU reference [24], we noted that the estimated median energy intake is in excess of recommended levels for all age groups. The observed high energy intakes in both groups is perhaps attributed by high amount of carbohydrates in the consumed diets. The carbohydrate content of the consumed diets contributed about 83, 78 and 75% of the total energy intakes for children aged 6–8, 9–11 and 12–23 months, respectively. Similarly, the calculated average energy densities of the consumed diets were high. A study by FANTA [12] on development of evidence based dietary recommendations for children and mothers living in western highlands of Guatemala revealed similar observations of higher energy intakes. Therefore, the present results suggest that if care is not taken, children may significantly suffer high prevalence of childhood overweight and obesity as indicated in Table 1 while at the same time being deficient on other essential nutrients.

We also noticed that the median protein intakes as well as dietary protein densities for all children are in the range of being adequate. However, the quality of protein may be inadequate considering that a large proportion of the protein in the diet was from maize and rice and that very few animal source foods were consumed. The quality of protein can positively and significantly affect the nutritional status of consumers [32]. This is clearly reflected in our study, where we observed a significant high prevalence of stunting despite higher levels of protein intakes in all study age groups (Table 2). Evidence from previous studies indicated that children who had high utilizable protein intakes were found to have a significantly lower prevalence of stunting [33–35]. These findings highlight the value of considering the quality of protein, when planning a nutrition intervention.

In the same way, we observed that the average intakes of vitamin A and C are in the range of being adequate. The high levels of vitamin A and C in the present study was to a great extent contributed by average breast-milk intake that was included in the analysis. These levels may not necessarily represent a real life situation in the study area because the analysis was based only on the published average intakes of breast-milk for each age group. The actual intakes of breast milk were not analysed, thus, we are not quite sure if the breast milk from mothers in the study population had a high content of these vitamins. One should note that maternal nutritional status can significantly affect the status of vitamin A and C in infants and young children [28, 36]. For that matter, if mothers had poor nutritional status, it is obvious that children would not achieve the nutrient recommendations for vitamin A and C which may significantly affect the linear growth of infants and young children [37, 38].

On the other hand, we observed that average intakes of either one or two or three of essential micronutrients such as iron, zinc and calcium are lower than the adequate ranges in all age groups. For example, according to the standard values established by WHO [25], the average intakes for iron are lower than the required adequacy ranges by 42 to 77% and the average intakes for zinc are lower than recommended intake by 40%. Likewise, the mean calcium intake is lower than RDA by 35 to 39%. Although iodine intakes were not measured in this study, its intake is likely to be lower than RDA because majority of households in the study area were observed to rely much on non-iodized salts in their diets. These results suggest that the content of iron, zinc and calcium in the consumed diets for each age group is suboptimal. Thus, it would be very difficult to meet the adequacy range of the stated micronutrients under existing feeding practices. The observed inadequacies of iron, zinc and calcium intakes are probably a contributing factor for the current high prevalence of stunting (Table 1) in the target population. Evidence from a previous study [38] indicates that stunting is accompanied by long term deficiencies of iron and other essential micronutrients. These findings suggest that access to nutrient rich formulations may be needed in this population to achieve sufficient iron, zinc and calcium intakes. However, as highlighted in the study by Skau and colleagues [13], food formulations should be integrated with approaches that encourage changes to traditional feeding practices for infants and young children.

Given the scope of the present study, our findings were based solely on cross-sectional data, which offers only a snapshot of a single moment in time. Furthermore, our analysis did not take seasonality into account. Thus, nutrient intakes and food consumption patterns identified refer essentially to the dry season in which data were collected. Further studies that extend beyond a single moment in time are therefore, needed to verify the effect of seasonality on food availability and consumption in the study population. Another limitation is that Tanzania food composition tables are not complete for all nutrients analysed in the present study. Any missing energy or nutrient contents were imputed from other nutrient composition databases, mainly the United States Department of Agriculture (USDA) food composition databases. The nutrient composition of foods is known to vary within and across regions and countries [15, 39], this might have affected our analysis, especially if the final nutrient composition of candidate foods is not accurate.

## Conclusions

The study shows that local foods in the study area have potential to achieve recommended dietary intakes of some essential nutrients, and that interventions are needed to meet the required amount of iron, zinc and calcium for children aged 6–23 months. The interventions we propose here may encourage changes in traditional feeding habits and practices of the target population. Possible intervention options are (1) supplementation of local foods with nutrient-dense foods that are not normally consumed in the locality (2) providing new avenues for increasing the production and wide consumption of local nutrient-dense foods, or optimizing the way local diets are constituted so as to achieve nutrient recommendations for infants and young children.

## Abbreviations

DRI: Dietary reference intake
EAR: Estimated average requirement
HAZ: Height-for-age z-score
RCH: Reproductive and child health
RDA: Recommended dietary allowance
RNI: Recommended nutrient intake
SSA: Sub-Saharan Africa
WAZ: Weight-for-age z-score
WDR: Weighed dietary record
WHZ: Weight-for-height z-score
